# *DEK* loss in HeLa cells does not alter genotoxic stress sensitivity and DNA damage response

**DOI:** 10.17912/micropub.biology.002200

**Published:** 2026-06-24

**Authors:** Xiaoxi Liu, Yewei Liu, Ferdinand Kappes

**Affiliations:** 1 Division of Natural and Applied Sciences, Duke Kunshan University, Kunshan, JS, China; 2 Department of Biosciences and Bioinformatics, Xi'an Jiaotong-Liverpool University, Suzhou, Jiangsu, China

## Abstract

DEK, a chromatin-associated oncoprotein, is involved in various cellular processes. Previous siRNA/shRNA-mediated *DEK* knockdown studies reported increased genotoxic stress sensitivity and altered γH2AX signaling. Whether *DEK* knockout cells recapitulate these phenotypes remains elusive. Using CRISPR/Cas9-generated *DEK* knockout HeLa cells, we assessed survival, γH2AX levels, and genomic integrity after DNA damage. *DEK* loss caused moderate, context-dependent changes in viability and damage accumulation. γH2AX induction and resolution were largely comparable between wild-type and knockout cells. Comet assays revealed moderate DNA damage upon DEK loss. Thus, complete *DEK* loss yields less pronounced DNA damage responses than knockdown, suggesting compensatory effects in HeLa cells.

**Figure 1. CRISPR-Cas9 mediated DEK knockout results in limited effects on DNA damage response and genotoxic stress sensitivity in HeLa cells:** ( **A** ) **Cell viability analyses:** HeLa WT and DEK KO cells were treated with increasing concentrations of doxorubicin (DOX, 0.1, 0.5, 2, 5 µM, 24 h), camptothecin (CPT, 0.1, 0.5, 2, 5 µM, 24 h), hydroxyurea (HU, 0.5, 1, 2, 5 mM, 48 h), or etoposide (ETOP, 20, 50, 100, 200 µM, 48 h). Cell viability was analyzed using CCK-8 and data were normalized to vehicle control (DMSO). Data are presented as mean ± SEM from three independent experiments (n = 3 per condition). ( **B** ) **Assessment of γH2AX abundance via Western blot analysis** : HeLa WT and DEK KO were treated with 2 µM DOX, 1 µM CPT, 1 mM HU, or 100 µM ETOP and total cell lysates from indicated time points were analyzed by western blotting using γH2AX -specific antibodies. H3-specific antibodies served as loading control. Data are presented as mean ± SEM from three independent experiments (n = 3). The four graphs depict the densitometric analysis of western blots shown in the panels below. Only one set out of three per condition is shown. ( **C** ) **Analysis of γH2AX abundance and distribution via confocal immunofluorescence:** Confocal images of γH2AX staining in HeLa WT and DEK KO cells following treatment with 2 μM DOX for 6 h were taken. Quantification of γH2AX fluorescence intensity (γH2AX) and spatial distribution (distribution of γH2AX ) showed variable and context-dependent differences between WT and KO cells. For each group, data is presented as mean ± SD. For the upper independent experiment, WT -, n = 82; KO -, n = 91; WT +, n = 81; KO +, n = 80. For the repeat, WT -, n = 130; KO -, n = 155; WT +, n = 206; KO +, n = 175. ( **D** ) **Assessment of genomic integrity via comet assay** : Neutral comet assays were performed using HeLa WT and DEK KO cells following treatment with 1 µM DOX for 24 h or 2 μM DOX for 6 h. DNA damage was quantified using tail moment. For each group, quantification of tail moment with SD is shown. For 1 µM DOX 24 h treatment, WT NT, n = 71; KO NT, n = 124; WT DOX, n = 124; KO DOX, n = 86. For 2 µM DOX 6 h treatment, WT NT, n = 61; KO NT, n = 143; WT DOX, n = 29; KO DOX, n = 98. Statistical comparisons between WT and KO cells for panels shown in A-D were performed using unpaired two-tailed Student’s t-test.

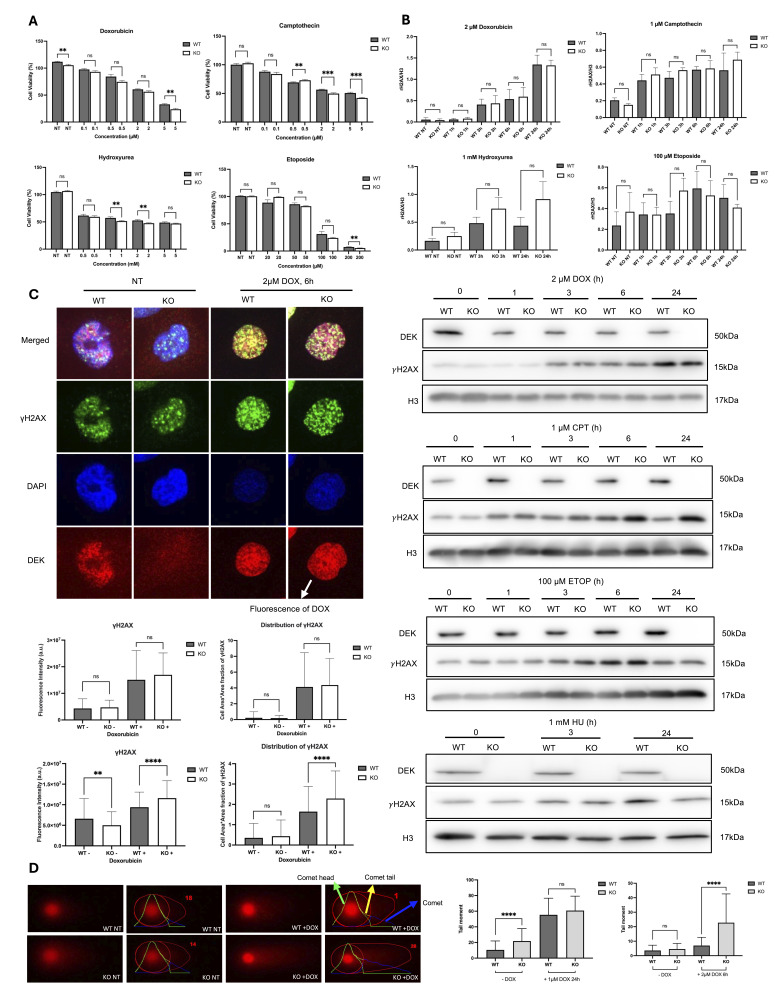



## Description

DEK is a highly conserved chromatin-associated protein involved, amongst others, in chromatin organization, DNA replication, and DNA repair (Alexiadis et al., 2000; Deutzmann et al., 2015; Peters et al., 2026; Pierzynska-Mach et al., 2023; Privette Vinnedge et al., 2013; Smith et al., 2017; Waldmann et al., 2002). Previous studies using siRNA- or shRNA-mediated DEK knockdown models reported that reduced DEK expression sensitizes cells to genotoxic stress, alters DNA repair capacity, and enhances DNA damage response (DDR) signaling, particularly through γH2AX-associated pathways (Kappes et al., 2008; Kavanaugh et al., 2011; Smith et al., 2017). However, whether complete genetic ablation of DEK produces similar effects remains unclear. Because knockout models eliminate residual DEK protein but may also permit long-term compensatory adaptation, we examined whether complete DEK ablation produces DDR phenotypes similar to those reported in DEK knockdown models.

First, and to determine whether complete DEK loss alters cellular sensitivity to genotoxic stress, cell viability was analyzed using Cell Counting Kit-8 (CCK-8) assays following treatment with doxorubicin (DOX), camptothecin (CPT), hydroxyurea (HU), and etoposide (ETOP). DEK KO cells exhibited moderate and dose-dependent changes in viability relative to wild-type (WT) cells ( **Figure 1A** ). Increased sensitivity was observed under specific high-dose DOX and CPT conditions, while responses to HU and ETOP were comparatively modest. Overall, although statistically significant differences were detected under several treatment conditions, complete DEK knockout produced modest effects on cell viability following treatment with DNA-damaging agents.

To further examine DDR activation, γH2AX protein levels were analyzed by western blotting following treatment with multiple DNA-damaging agents. As expected, γH2AX levels increased over time following exposure to DOX, CPT, HU, and ETOP in both WT and KO HeLa cells ( **Figure 1B** ). However, quantitative analyses demonstrated that overall γH2AX induction remained largely comparable between cell types across most conditions examined. These findings contrast with previous knockdown-based studies reporting stronger DDR defects following DEK depletion (Deutzmann et al., 2015; Kappes et al., 2008; Kavanaugh et al., 2011; Smith et al., 2017).

To evaluate γH2AX signaling at the single-cell level, immunofluorescence analyses were performed following DOX treatment ( **Figure 1C** ). In one independent experiment, γH2AX fluorescence intensity and distribution did not significantly differ between WT and KO cells. In a second independent experiment performed under identical conditions, DEK KO cells exhibited increased γH2AX intensity and a more dispersed γH2AX distribution pattern relative to WT controls. However, these phenotypes were variable and not uniformly reproducible across experiments or treatment conditions. Together, these findings suggest that complete DEK loss may modestly affect spatial organization of DDR-associated chromatin signaling without robustly altering overall γH2AX accumulation.

To directly assess DNA damage accumulation, comet assays were performed following genotoxic stress treatments ( **Figure 1D** ). DEK KO cells exhibited increased tail moment values relative to WT cells following treatment with 2 μM DOX for 6 h, indicating elevated DNA damage accumulation under specific conditions. However, the magnitude of these differences varied between independent experiments. In contrast, treatment with 1 µM DOX for 24 h increased overall DNA damage in both genotypes without producing statistically significant differences between WT and KO cells. Overall, complete DEK loss produced relatively moderate and drug concentration-dependent effects on DNA damage accumulation.

Collectively, these findings indicate that complete DEK knockout in HeLa cells produces modest DDR-associated phenotypes as compared to data from previously reported DEK knockdown models. Although measurable differences between WT and KO cells were detected under certain experimental conditions, these effects were generally modest and variable. One possible explanation is that stable genetic ablation of DEK permits long-term compensatory cellular adaptations that are less likely to occur during transient knockdown. Alternatively, residual DEK expression in knockdown systems may produce distinct chromatin perturbations compared with complete protein loss. Overall, these findings suggest that phenotypes observed in transient DEK depletion systems may not fully reflect the consequences of stable genetic DEK ablation.

## Methods

Cell culture and DEK knockout

Matched HeLa WT and DEK KO cells were obtained from Ubigene Biosciences. Specifically, the KO was engineered using the CRISPR/Cas9 system targeting the early coding region. Successful genomic editing was validated by Sanger sequencing of the target locus, which confirmed the presence of a frame-shift mutation starts at the 8th amino acid. Both cell lines were maintained in high-glucose Dulbecco’s Modified Eagle Medium (DMEM; Adamas life, C8060-500 mL) supplemented with 10% fetal bovine serum (FBS; Cellterlife, Cat# CLPFBS500) and 1× penicillin-streptomycin (Biosharp, Cat# BL505A), and incubated in a humidified cell culture incubator at 37°C with 5% CO _2_ .

Drug treatment

For Cell Counting Kit-8 assays, cells were treated with increasing concentrations of Doxorubicin (Beyotime, Cat# SC0141) for 24h, Camptothecin (Beyotime, Cat# SC0141) for 24h, Hydroxyurea (Beyotime, Cat# S961-1g) for 48h, and Etoposide (Beyotime, Cat# SC0173) for 48h. Drugs in powder form were dissolved in dimethyl sulfoxide (DMSO, Biosharp, Cat# BL165B). Specific concentration and treatment of time points were selected for Comet Assays, Western blotting, and Immunofluorescence experiments as indicated in the respective figures.

Cell viability assay

Cell viability was assessed using the Cell Counting Kit-8 (CCK-8; Beyotime, Cat# C0038; Adamas Life, Cat# C8022). Cells were seeded at 2,000 cells per well in 96-well plates and incubated for 24h prior to drug treatment. Cells were then treated with increasing concentrations of DOX (0.1 µM, 0.5 µM, 2 µM, 5 µM) or CPT (0.1 µM, 0.5 µM, 2 µM, 5 µM) for 24h, or HU (0.5 mM, 1 mM, 2 mM, 5 mM), or ETOP (20 µM, 50 µM, 100 µM, 200 µM) for 48h. Following treatment, CCK-8 reagent was added to each well and incubated for 4h at 37°C. Absorbance was measured at 450 nm using a microplate reader. Cell viability values were normalized to the vehicle (DMSO) control group.

Western blot analysis

Total cell lysates were prepared using 2% SDS followed by determination of total protein concentration via a BCA Protein Assay Kit (Beyotime, Cat# P0010). Equal amounts of protein (20 µg) were separated by 4-20% SDS-PAGE (typically 12%) and transferred onto PVDF membranes (Immobilon-PSQ, MilliporeSigma, Cat# ISEQ00010). Membranes were blocked with 5% skim milk in TBS-T for 1h at RT and then incubated with phospho-histone H2AX (Ser139) rabbit polyclonal antibodies (1:1000 dilution, Beyotime, AF5836), histone H3 mouse monoclonal antibodies (1:1000 dilution, Beyotime, AF0009), DEK (K-877, 1:10,000 dilution; in-house) (Kappes et al., 2004), and GAPDH rabbit monoclonal antibodies (1:1000 dilution, Beyotime, AG8015) for 2h at RT. After washing three times with TBS-T for 10 min each, membranes were incubated with horseradish peroxidase-conjugated (HRP) goat anti-rabbit (Beyotime, A0208) or HRP goat anti-mouse secondary antibodies (Beyotime, A0216) for 1h at RT. Following three washes with TBS-T for 10 min each time membranes were exposed to enhanced chemiluminescence reagents and bands were detected by chemiluminescence using Amersham ^TM^ ImageQuant ^TM^ 800 imaging system. Band intensities were quantified using ImageJ software, and normalized to histone H3 levels, expressed as ratio of γH2AX to H3.

Immunofluorescence confocal microscopy

Cells were grown on poly-L-lysine-coated coverslips placed in 6-well plates at 3x10 ^5^ cells per well prior to drug treatment. After mock treatment (NT) or drug treatment with 2 µM DOX for 6h, cells were washed with PBS three times, followed by a fixation step with 4% paraformaldehyde for 15 min at RT. Cells were permeabilized with 0.6% Triton X-100 for 5 min at RT, washed with PBS three times for 5 min each, blocked with 4% BSA and 0.1% TritonX-100 blocking buffer (in PBS) for 30 min at RT, and incubated with phospho-histone H2AX (Ser139) rabbit polyclonal antibody (1:1000 dilution, Beyotime), and purified mouse anti-human DEK antibody (1:1000 dilution, BD Biosciences) in the dilution buffer (1% BSA in 1x PBST) for 1h at RT. After washing with PBS for three times, each for 5 mins, cells were incubated with Alexa Fluor 488-conjugated (goat anti-rabbit, Beyotime, A0423) and Alexa Fluor 555-conjugated (donkey anti-mouse, Beyotime, A0460) IgG for 30 min at RT (1:500 dilution) in the dark. After washing, coverslips were mounted on the slides with antifade mounting medium containing DAPI and stored in the dark at 4°C.

Confocal images were acquired using a laser-scanning confocal microscope (Olympus FluoView FV3000) equipped with a 60× super-resolution objective. Fluorescence signals were sequentially captured using the 405 nm channel (DAPI), 488 nm channel (γH2AX), and 561 nm channel (DEK). Imaging parameters were kept consistent across all experimental conditions to ensure comparability. For each sample, at least 50 cells were imaged and included in the analysis. Image analysis was performed using ImageJ. Both manual quantification and a semi-automated algorithm optimized for immunofluorescence image analysis were applied to ensure robustness and reproducibility. The mean nuclear fluorescence intensity of γH2AX and the cell area times area fraction of γH2AX foci per nucleus were used as two quantitative metrics. Statistical analysis was conducted using an unpaired two-tailed Student’s t-test. Data are presented as mean ± SD, and a P < 0.05 was considered statistically significant.

Comet assay

DNA strand breaks were assessed using neutral comet assays following established protocols (Olive & Banath, 2006). Briefly, a 0.5 M Na₂EDTA stock solution (pH 8.0) was prepared by dissolving 55.8 g Na₂EDTA and 6.4 g NaOH in 270 mL of distilled water with continuous stirring for approximately 2 h, followed by adjustment of the pH to 8.0 with additional NaOH. A 5 M NaOH stock solution was prepared by slowly dissolving NaOH pellets in ice-cold distilled water. For neutral comet assays detecting DNA double-strand breaks, the neutral lysis buffer contained 2% sarkosyl, 0.5 M Na₂EDTA and 0.5 mg/ml proteinase K (pH 8.0). The neutral electrophoresis buffer consisted of 90 mM Tris, 90 mM boric acid and 2 mM Na₂EDTA (pH 8.5).

Cells were seeded at 3 × 10⁵ cells per well in 6-well plates and incubated for 24 h before treatment. Then, cells were treated with either 1 µM DOX for 24 h or 2 µM DOX for 6h. After treatment, cells were harvested and diluted to 4 × 10⁴ cells/ml in PBS. Cell suspensions were mixed with 1% low-melting point agarose and spread onto microscope slides. After agarose solidification, slides were gently immersed in the appropriate lysis buffer. For neutral comet assays, slides were incubated overnight in neutral lysis buffer at 37°C. Slides were subsequently washed three times in neutral electrophoresis buffer for 30 min each and electrophoresed for 25 min at 0.6 V/cm. After electrophoresis, slides were rinsed with distilled water and stained with propidium iodide (10 µg/mL) for 20 min in the dark. At least 50 nuclei per sample were imaged using an inverted fluorescence microscope at 4x magnification.

Tail DNA percentage, tail length, tail moment (tail intensity multiplied by tail length), and olive moment (% of DNA within the tail multiplied by the distance between the centers of the head and tail) were quantified using OpenComet, an ImageJ plugin. Tail moment was used as the primary parameter for statistical comparison between groups. Statistical analyses were performed using GraphPad Prism. Differences between groups were evaluated using unpaired two-tailed Student’s t-tests assuming Gaussian distribution. P values < 0.05 were considered statistically significant.

Statistical analysis

Statistical analyses were performed using GraphPad Prism. Differences between groups were evaluated using unpaired two-tailed Student’s t-tests. Data are presented as mean ± SD or SEM as indicated in the figure panels. P values < 0.05 were considered statistically significant.
